# Optimizing mung bean productivity and root morphology with biofertilizers for sustainable farming

**DOI:** 10.1038/s41598-025-28815-8

**Published:** 2025-11-18

**Authors:** Afsaneh Yousefi, Jaafar Nabati, Reza Mirzaeetalarposhti, Ali Malakshahi Kurdestani

**Affiliations:** 1 Institute of Crop Science, University of Hohenheim, 70599 Stuttgart, Germany; 2 Department of Agrotechnology, Faculty of Agriculture, Ferdowsi University of Mashhad, Mashhad, Iran

**Keywords:** Mung bean, Biofertilizer, Nodule activity, Sustainable agriculture, Total root length, Plant ecology, Plant physiology, Rhizobial symbiosis

## Abstract

The excessive use of chemical fertilizers has raised major environmental and economic concerns in legume cultivation. This study assessed the effects of various fertilizers, including biofertilizers and chemical nitrogen, on yield, root characteristics, and nutrient dynamics in two mung bean genotypes (Partow, IC418452). Field trials were conducted over a two-year period in Mashhad, Iran, using a factorial randomized block design. Treatments included two genotypes and six fertilizer levels: control, N-fixing bacteria (FLNF), P-solubilizing bacteria (PSB), K-solubilizing bacteria (KSB), a consortium (FLNF + PSB + KSB), and Urea. Yield components, biomass, root morphology, nodulation, and plant/soil NPK concentrations were measured and analyzed. Fertilizers significantly affected yield, biomass, root structure, and nutrient uptake. Urea yielded the highest biomass, grain yield, and root area, especially in Partow. However, the microbial consortium significantly improved yield components compared to the control and uniquely maximized root nodulation and inoculation percentage, indicating an enhanced biological nitrogen fixation potential. On average across the two seasons, urea increased grain yield by 46% and biomass by 41% relative to the control, whereas the microbial consortium enhanced root nodulation by 62% and yield by 32%. Significant genotype×fertilizer interactions highlighted genotype-specific responses. Nodulation correlated positively with yield. Both urea and the PGPR consortium significantly increased mung bean productivity. While urea maximized yield, the consortium provided considerable yield gains and enhanced biological nitrogen fixation potential, presenting a viable and sustainable alternative to reduce reliance on chemical nitrogen. The goal was to understand the relationships between root morphology, nutrient utilization, and yield to promote sustainable, high-yield mung bean cultivation. These findings highlight the potential of multi-strain biofertilizers to maintain mung-bean productivity while reducing dependence on synthetic N inputs.

**Supplementary Information:**

The online version contains supplementary material available at 10.1038/s41598-025-28815-8.

## Introduction

Leguminous crops are vital components of sustainable agricultural systems due to their ability to fix atmospheric nitrogen and enhance soil fertility. Among them, mung bean (*Vigna radiata* L. Wilczek) is valued for its short growth cycle, high-quality protein, and capacity to restore degraded soils^1,2^. The capability of biological N fixation, production of highly digestible forage, and the short growth period are prime advantages of the mung bean for its inclusion in crop rotations in different regions^3,4^. Approximately 175 million tons of nitrogen (N) per year are estimated to be added to soils through biological N fixation^5^, which contributes to system sustainability, grain quality for human or livestock consumption, and soil fertility^6^.

High inputs of chemical N, P, and K fertilizers have been globally employed to increase productivity and plant growth^7^. However, the excessive application of chemicals K, P, and N has resulted in profound negative changes in soil characteristics and the environment^8^. Therefore, it is time to identify alternative methods to ensure competitive crop yields and environmental health while maintaining a sustainable agroecosystem^9^. Although legumes can meet a large share of their nitrogen demand through biological fixation, they still need a small starter amount of available N to support early vegetative growth until an effective rhizobial symbiosis is established. Application of inoculants formed of plant-growth-promoting rhizobacteria (PGPR), which include a set of helpful soil bacteria that associate with plants, contributing to the overall strength of the plants, tolerance to abiotic and biotic stresses, water and nutrient uptake, and enhancing root expansion^10–12^. Bacterial species such as *Bacillus* sp., *Enterobacter* sp., *Azospirillum*, *Azotobacter*, *Thiobacillus*, and *Pseudomonas*, among others, have been shown to act as PGPR in association with various plant species^13^; therefore, PGPR deserves particular attention in agriculture.

Furthermore, in addition to positively affecting yield, some strains can also suppress the growth of plant pathogens^14^. The beneficial effect of *Azospirillum* and *Azotobacter* may derive from their biological N fixation and stimulating impact on root growth^15^. The root system exerts a range of functions, including carboxylate production, water uptake, nutrient absorption, and interaction with soil microbes^16^. Root morphology and physiology strongly affect aboveground growth and development^17^. Applying PGPR optimizes the morphology and distribution of roots, promoting plant N absorption and resulting in enhanced yield and nutrient uptake^18,19^. Bashan et al. (2014) demonstrated the effectiveness of this inoculant on various plants, showing its ability to promote root growth through auxin production and enhance N supply via nitrogenase activity^20^.

The shifts in the C/N ratio and modifications in the root structure (21). PSB (P-solubilizing bacteria) and KSB (K-solubilizing bacteria) (may improve crop nutrient absorption through solubilizing insoluble P and releasing K from silicate in soil^22,23^. It was reported that biofertilizers in beans (*Phaseolus vulgaris* L.) improved N and P uptake and increased germination percentage, plant height, 100-seed weight, and dry matter^24,25^. In addition, plant-growth-promoting rhizobacteria (PGPR), such as Bacillus, Paenibacillus, and Enterobacter species, can solubilize insoluble phosphorus and potassium, synthesize phytohormones, and enhance water and nutrient uptake^11,26^. These multifaceted functions make PGPR an important tool for sustainable nutrient management.

Previous research has primarily examined individual inoculants, whereas studies integrating multi-strain PGPR consortia with conventional nitrogen sources are scarce. Recent evidence shows that combining microbial inoculants can increase nutrient availability and resilience under abiotic stress^27–29^. However, the extent to which such consortia affect mung-bean root morphology, nutrient uptake, and yield—particularly across different genotypes—remains unclear. Therefore, the present study aimed (I) to investigate the effects of different fertilizers on yield and yield components, nutrient uptake (II) to compare root morphologies (total root length, root volume, root area, and Number of nodules per root), and (III) to observe the effect of different fertilizers on soil indicators. Such an experiment would shed light on the relationship between root morphology, nutrient concentration, and yield and provide a theoretical and practical foundation for high-yielding cultivation with PGPR application.

## Materials and methods

**Fig. 1:**
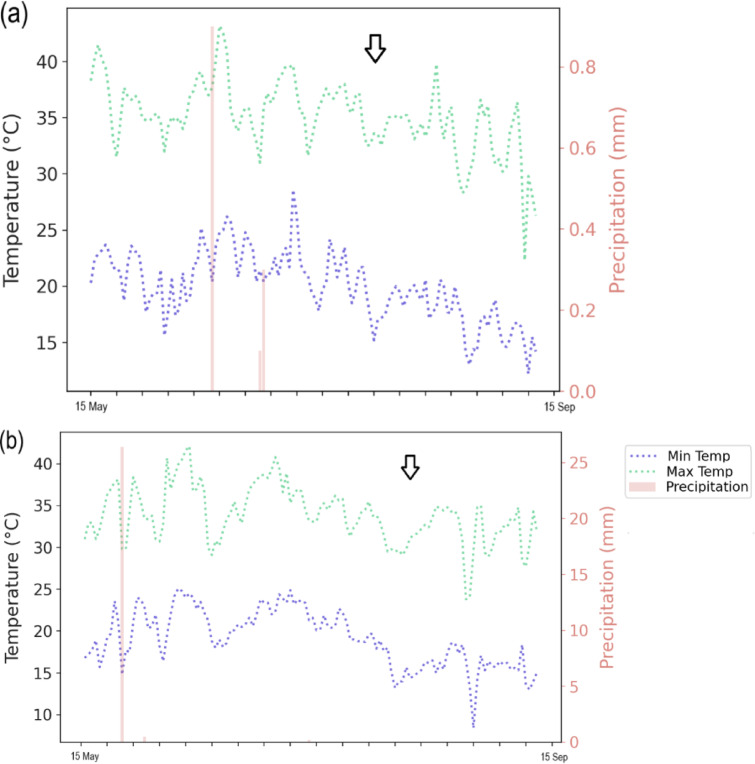
Maximum and minimum daily temperature and daily precipitation in 2021 (a) and 2022 (b) during the growing seasons of mung bean. Arrowhead means flowering time.

Field studies were conducted in Mashhad, Iran, in 2021 and 2022 (latitude: 36°15′N, longitude: 59°28′E, altitude: 985 m, and mean annual rainfall of 30 years: 227.8 mm). Table 1 provides details of the experimental soil at the sites. The daily temperatures for 2021 and 2022, along with the long-term site averages obtained from the meteorology site, are shown in Fig. 1.

**Table 1:** Experiment site details for the 2021 and 2022 mung bean experiments.

Year	Soil pH (CaCl_2_)	Electrical conductivity (dS.m^− 1^)	Texture	C_min_ (%)	C_org_ (%)	*N*_tot_ (%)	*P* (mg.kg^− 1^)	K(mg.kg^− 1^)
2021	7.41	1.18	Loam	1.65	0.61	0.15	90.4	189.5
2022	7.71	1.01	Loam	1.75	0.65	0.16	94.3	196.2

The study employed a factorial, completely randomized block design with three replicates. Genotype was in two levels^1^: Partow^2^, IC418452. Partow is a local Iranian cultivar that was registered by the Iranian Seed and Plant Improvement Institute (SPII) and the Research Institute for Registration and Certification of Seeds and Seedlings (RIRCSS) in 1994. Additionally, IC418452, registered in 2003, is known as a tolerant variety against abiotic stresses^30^. The experiment was performed in two years, 2021 and 2022, as one of the factors. Biofertilizers were used in six levels^1^: FLNF (Free-living N fixing bacteria)^2^, PSB (Phosphate solubilizing bacteria)^3^, KSB (Potassium solubilizing bacteria)^4^, microbial consortium (FLNF + PSB + KSB)^5^, N (Urea), and^6^ control (without biological and chemical fertilizers). Each liquid biofertilizer contains 109 colony-forming units (CFU) per milliliter^31^, manufactured by the Dayan Company in Iran (Table 2).

**Table 2:** The supplementary bacterial content of biofertilizer used in this study.

Biofertilizers		
Free-living nitrogen-fixing bacteria (FLNF) DAYAN Nitro-Bacter^®^	Phosphate solubilizing bacteria (PSB) Phospho Bacter Dayan^®^	Potassium-solubilizing bacteria (KSB) DAYAN Petas Bacter^®^
*Bacillus subtilis* NCBI Accession No. MT102419	*Paenibacillus polymyxa* NCBI Accession No. MT102424	*Enterobacter hormaechei* NCBI Accession No. MT102420
*Enterobacter cloacae* NCBI Accession No. MT102416	*Bacillus pumilus* NCBI Accession No. MT102425	*Enterobacter* sp. NCBI Accession No. MT102421
*Bacillus cereus* NCBI Accession No. MT102418	-	-

The experimental plot measured 4 m × 3 m, with a 50 cm distance between rows (six rows per plot), and a 7 cm distance between plants within a row. Seeds were surface-sterilized with 1% sodium hypochlorite, rinsed, and coated with the designated biofertilizer (50 mL kg⁻¹ seed) immediately before sowing. Seeds were planted by hand on May 14, 2021, and May 28,2022. Bacterial treatments were applied in three stages^1^: at the time of sowing as seed coating^2^, with the second irrigation, and^3^ before the flowering stage. Urea was applied to 40 kg. ha^− 1^in two stages^1^: before sowing (7 May) and^2^ before flowering (16 June) for the N treatment through solving in the irrigation water. The experimental plots were irrigated once the seeds were planted, and subsequent irrigations occurred every 7–10 days based on climate and plant demands. Weeds were manually removed twice during the growing season—at 20 and 40 days after mung bean emergence. Pest control was performed using integrated pest management practices that excluded the use of systemic insecticides.

### Determination of N, P, and K concentration

The samples were analyzed to determine the concentrations of N, P, and K in the soil and above-ground plant parts. By grinding the plant dry matter, the mineral nutrient status of the dried plant material was determined using an established wet-chemical extraction method (VDLUFA, 2011). P concentration in the plant extraction was determined at 436 nm using vanadate/molybdate as a color agent in the spectrophotometer (Visible-UV Jenway Model Spectrophotometer 6305, USA). K was measured using flame emission photometry (Jenway PFP7, England) (Jackson, 1973). For nitrogen concentration determination, the sample will be digested in concentrated H2SO4 within a digestion mixture of copper sulfate, potassium sulfate, and mercuric oxide. Soil samples are also analyzed to measure nitrogen (N), phosphorus (P), and potassium (K). The Kjeldahl method will measure nitrogen concentration (Piper, 1966). To determine the P and K concentrations in soil samples, the samples will be digested with a diacid mixture of HNO_3_ and HClO_3_.

### Root morphology

To study root morphology, plants were harvested, and their roots were gently rinsed with distilled water. The roots were scanned at 300 dpi by Delta T Scan. The Image Analysis, using Delta-T Devices, analyzed the morphometric parameters (total root length and root area). To calculate the number of nodules at the flowering stage of mung bean, five plants were harvested entirely with their root system by a hoe at a soil depth of 50 cm. Active nodules were identified by their pink coloration, and the total number of nodules per plant was counted. The analysis method followed Nabati et al. (2025).

### Final harvest

Whole plants (two genotypes × six fertilizers × three replicates = 36 plots) were harvested at physiological maturity, defined as when 95% of the pods were brown. Subsequently, the following variables were measured: plant height, number of pods per plant, 100-seed weight, and yield.

### Statistical analysis

The combined analysis of variance was carried out with ‘genotypes’ and ‘fertilizers’ as fixed effects and ‘year’ as a random effect using the General Linear Model (GLM procedure of the Statistical Analysis System). SAS v. 9.1; SAS Institute Inc., Cary, NC, USA, was used to analyze variance, compare means using LSD tests, and calculate correlation coefficients between traits. JMP 4.0 and GraphPad Prism v. 9 software were also used for graphical analysis.

## Result

### Interactive effects on Biomass, Yield, and morphological traits

**Fig. 2:**
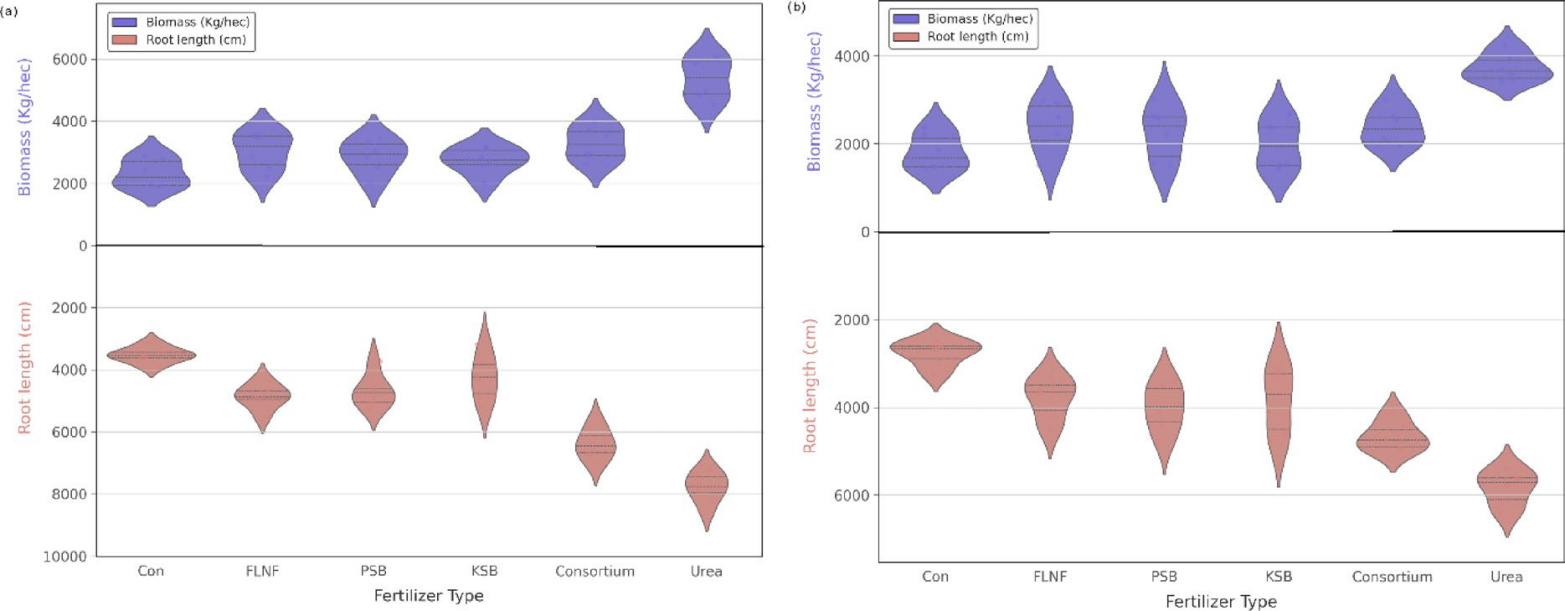
Mean comparison of biomass and root length of mung bean interaction affected by fertilizers and genotypes under different fertilizers (a) Partow, (b) IC418452 (data averaged over two years). The error bars on the graph represent a 95% confidence interval (CI).

**Fig. 3:**
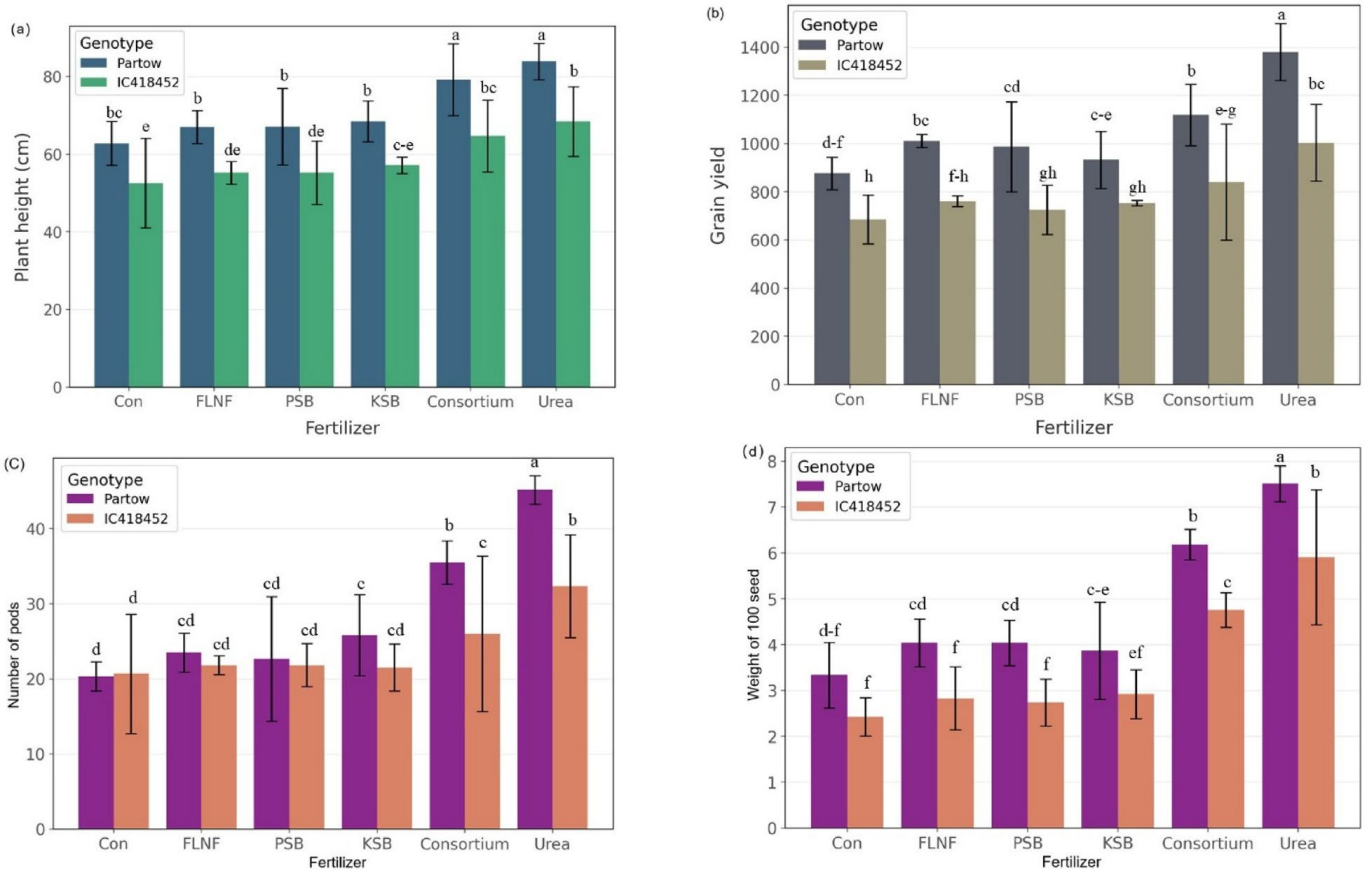
Mean comparison of plant height (a), Grain yield (b), Number of pods per plant (c), and weight of 100 seeds (d) of mung bean interaction affected by fertilizers and genotypes under different fertilizers (data averaged over two years). The error bars represent 95% confidence intervals (CI).

The combined ANOVA results (Table 3) showed that year, genotype, and fertilizer had highly significant effects (*p* < 0.001) on biomass, grain yield, 100-seed weight, plant height, and the number of pods per plant. The main effects of the year (Y), genotype (G), and fertilizer (F) were highly significant (*p* < 0.001) for biomass, grain yield, and weight of 100 seeds. Across both seasons, the highest biomass and grain yield were obtained with urea fertilization (6,245 kg ha⁻¹ and 2,145 kg ha⁻¹, respectively), followed by the microbial consortium (5,870 kg ha⁻¹ and 1,965 kg ha⁻¹, respectively). Partow produced higher biomass (6,245 kg ha⁻¹) and grain yield (2,145 kg ha⁻¹) than IC418452 (5,420 kg ha⁻¹ and 1,825 kg ha⁻¹, respectively). For biomass, a significant interaction between genotype and fertilizer (G × F, *p* < 0.001) was observed, indicating that the response to fertilizer varied across genotypes. Partow exhibited the highest biomass in the second year with urea application (6,540 kg ha⁻¹) (Fig. 2). Grain yield exhibited significant Y × G, Y × F, and G × F interactions (*p* < 0.001), showing that Y and G modified fertilizer effects. Partow with urea in the second year produced the highest yield (2,310 kg ha⁻¹) (Fig. 3b). Yields were 15–30% higher in 2022 across most treatments (Table S1, Fig. 1). For plant height, only the main effects of year, genotype, and fertilizer were significant (*p* < 0.001), with no notable interactions, implying consistent effects across all factor levels. Partow in the second year showed the most remarkable plant height (71.5 cm); there were significant differences between fertilizers, but not between urea and microbial consortium (70.8 and 69.5 cm, respectively) (Fig. 3a).

**Table 3:** Combined analysis of variance of the main and interaction effects of genotypes and different fertilizers on the mung bean plant growth indicators over two years. Significance levels (* *p* < 0.05, **p < 0.01, ***p < 0.001) and Ns for not significant.

S.O.V	df	Biomass	Grain yield	Weight of 100 seeds	Plant height	Plant pods
Year (Y)	1	12,620,773***	613,484***	4.57***	2560.52***	975.35***
Rep × Y	4	45,839 ns	4484 ns	0.38*	20.17 ns	4.22 ns
Fertilizer (F)	5	9,284,686***	258,941***	27.7***	626.77***	587.71***
Y × F	5	19,711 ns	22,326***	0.54**	27.64 ns	117.11***
Genotype (G)	1	12,756,608***	1,184,028***	27.45***	2821.64***	415.68***
Y × G	1	43,752 ns	27,235***	16.18***	53.86 ns	13.34 ns
G × F	5	500,500***	15,104***	0.22 ns	12.31 ns	83.25***
Y × G × F	5	98,585 ns	4366 ns	0.07 ns	9.2 ns	12.44 ns
Error	44	57,663	2156	0.11	13.08	5.9
CV%		5.9	10.0	7.2	11.1	6.5

Pod number showed significant Y × F and G × F interactions (*p* < 0.001), with Partow producing 28.8 pods per plant versus 24.02 in IC418452 over two years (Fig. 3c). No Y × G × F interaction was significant for any trait. For 100-seed weight, significant Y × G (*p* < 0.001) and Y × F (*p* < 0.01) interactions indicated year-dependent genotype and fertilizer effects; the highest value occurred in Partow with urea in the second year (7.9 g) (Fig. 3d).

### Root development and nodulation traits

The main effects of genotype (G) and fertilizer (F) were highly significant (*p* < 0.001) for root area and root length, whereas year (Y) was not significant (Table 4). For the root area, significant interactions included Y × G (*p* < 0.05), Y × F (*p* < 0.001), and G × F (*p* < 0.001), indicating that the effects of genotype and fertilizer varied by year and in combination with each other. Root area was higher in 2021 for urea but more consistent across biofertilizers in 2022 (Table S2). The highest root area (486.97 cm² plant⁻¹) and root volume (141.67 cm³ plant⁻¹) were recorded for Partow under urea, while the lowest were in the control (127.70 cm² plant⁻¹ and 70.85 cm³ plant⁻¹, respectively).

**Table 4:** Combined analysis of variance of the main and interaction effects of genotypes and different fertilizers on root architecture and nodulation of mung bean over two years. Significance levels (*p < 0.05, **p < 0.01, ***p < 0.001) and Ns for not significant.

S.O.V	df	Root area	Root length	Root volume	No. of root nodules per plant	Inoculation (%)
Year (Y)	1	62 ns	130,588 ns	2241.33***	220.5***	31.73*
Rep × Year	4	1892 *	139,842 ns	44.62 ns	4.41 ns	22.67 ns
Fertilizer (F)	5	161,663***	19,743,439***	5227.56***	241.03***	333.01**
Y × F	5	4554***	69,823 ns	66.37*	13.1**	10.9 ns
Genotype (G)	1	65,475***	21,598,762***	7264.72***	304.22***	963.6**
Y × G	1	3621*	5,347,479***	577.64***	107.55***	43.24 ns
G × F	5	4868***	1,128,896***	353.66***	24.62***	22.63 ns
Y × G × F	5	962 ns	381,260*	44.08 ns	12.42**	8.9 ns
Error	44	565	143,860	23.98	2.72	10.35
CV%		7.3	5.6	11.0	8.1	8.9

For IC418452, the root area ranged from 100.88 cm² plant⁻¹ under control to 359.10 cm² plant⁻¹ under urea, and the root volume varied between 56.24 cm³ plant⁻¹ (control) and 100.51 cm³ plant⁻¹ (urea). Similarly, root length showed significant interactions for Y × G (*p* < 0.001) and G × F (*p* < 0.001), as well as a marginally significant three-way interaction, Y × G × F (*p* < 0.05). The most extended root length was found in Partow with urea (7771.30 cm), followed by Consortium (6377.55 cm), whereas the shortest occurred in the IC418452 control (2755.77 cm).

The number of root nodules per plant exhibited significant main effects for all factors (*p* < 0.001) and significant interactions, including Y × G (*p* < 0.001), Y × F (*p* < 0.01), G × F (*p* < 0.001), and Y × G × F (*p* < 0.01). Nodulation increased by 20–40% 2022 under consortium treatment, reflecting favourable climatic conditions (Table S2, Fig. 6). In the second year, Partow and the microbial consortium exhibited the highest number of nodules per plant (26.5). In contrast, the lowest was in the IC418452 control (6.8).

**Fig. 4:**
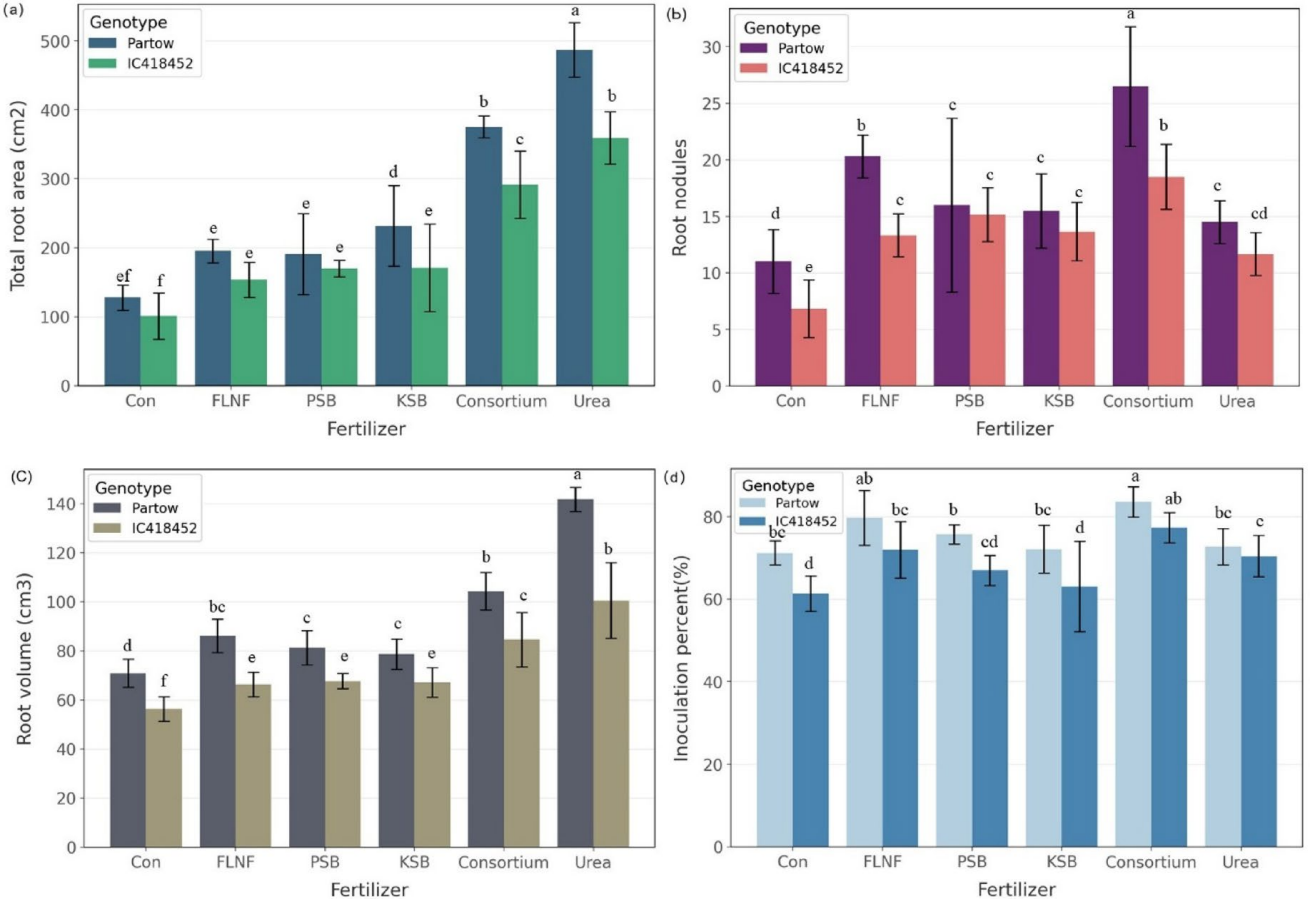
Mean comparison of root area (a), number of root nodules per plant (b), root volume (c), and nodule inoculation percent (d) of mung bean interaction affected by fertilizers and genotypes under different fertilizers (data averaged over two years). The error bars on the graph represent a 95% confidence interval (CI).

**Fig. 5:**
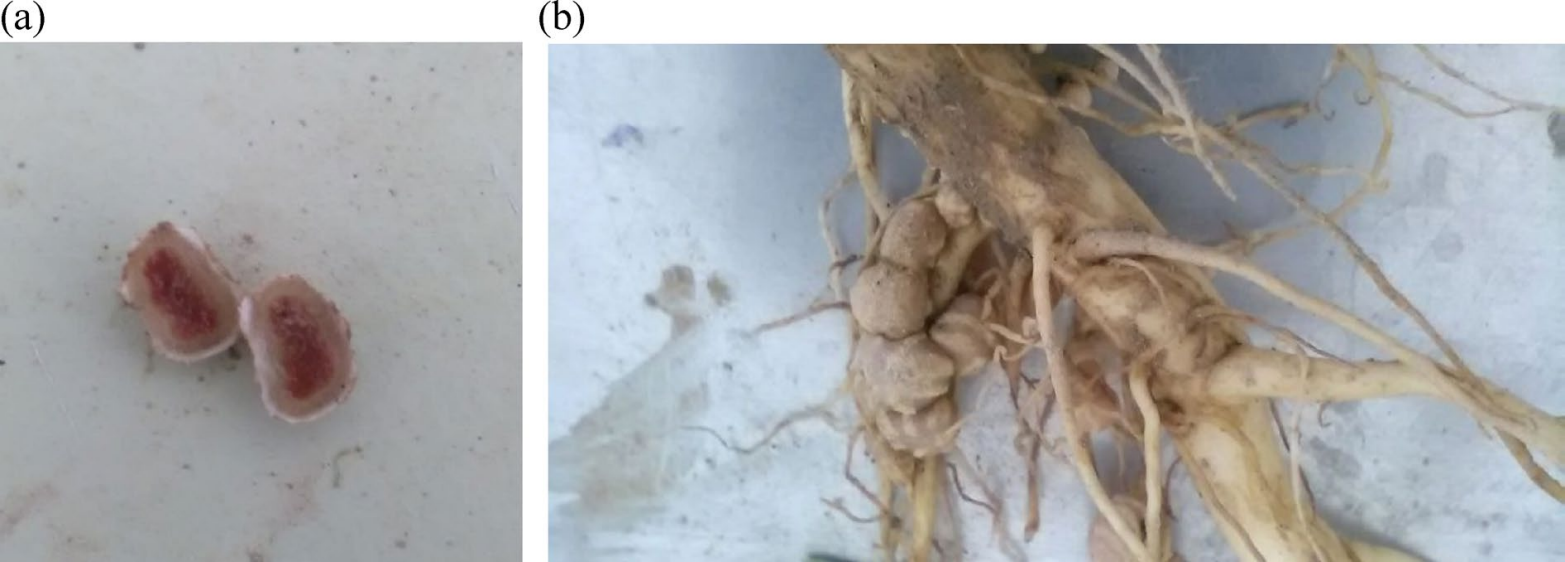
(a) A close-up view of active nitrogen-fixing nodules on mung bean roots, and (b) A detailed view of the nodules’ positioning along the root (Photos: Afsaneh Yousefi).

For root volume, all main effects—year, genotype, and fertilizer—were highly significant (*p* < 0.001), with significant interactions for Y × G (*p* < 0.001), Y × F (*p* < 0.05), and G × F (*p* < 0.001). The control treatment showed the lowest root volume (70.85 cm³ plant⁻¹ for Partow and 56.24 cm³ plant⁻¹ for IC418452) compared with the others (Fig. 4c). In contrast, inoculation percentage had significant main effects for year (*p* < 0.05), genotype (*p* < 0.01), and fertilizer (*p* < 0.01), but no significant interactions, suggesting independent effects of the factors on this trait. Partow and the microbial consortium exhibited the highest percentage of inoculation (83.6%), followed by IC418452 under the same treatment (77.4%). Pink nodules are active nodules, and the differences between inoculated and non-inoculated roots are illustrated in Fig. 5.

### Shoot and seed nutrient content

The main effects of year (Y), genotype (G), and fertilizer (F) were significant for N content per plant (Y: *p* < 0.05; G: *p* < 0.001; F: *p* < 0.001), with significant interactions for Y × F (*p* < 0.05), G × F (*p* < 0.05), and Y × G × F (*p* < 0.05), indicating that fertilizer effects on shoot nitrogen content varied by year and genotype, with a combined three-way influence (Table S3). The highest N content was recorded for ‘Partow’ under urea application in the second year (Fig. S1a).

All main effects were highly significant for shoot P content (*p* < 0.001). The Y × F interaction (*p* < 0.001) was significant, showing that the fertilizer’s impact on phosphorus content differed across years, whereas G × F and Y × G × F were not significant (*p* > 0.05). The lowest shoot P content was observed in the control and KSB treatments, ranging from 4.63 mg plant⁻¹ (Partow control) and 4.31 mg plant⁻¹ (Partow KSB) to 3.52 mg plant⁻¹ (IC418452 control) and 3.65 mg plant⁻¹ (IC418452 KSB). The highest values were recorded under urea (10.66 mg plant⁻¹ for Partow and 9.51 mg plant⁻¹ for IC418452) and consortium treatments (7.50 mg plant⁻¹ for Partow and 6.55 mg plant⁻¹ for IC418452) (Fig. S1b).

Similarly, shoot K content showed highly significant main effects (*p* < 0.001) but no significant interactions (all *p* > 0.05), indicating that year, genotype, and fertilizer had independent effects on potassium content. The lowest shoot K content was observed in KSB, PSB, and control treatments, ranging from 19.61 mg plant⁻¹ (Partow control), 20.77 mg plant⁻¹ (Partow PSB), and 25.09 mg plant⁻¹ (Partow KSB) to 16.84 mg plant⁻¹ (IC418452 control), 16.93 mg plant⁻¹ (IC418452 PSB), and 20.25 mg plant⁻¹ (IC418452 KSB). The highest K contents occurred with urea (40.71 mg plant⁻¹ for Partow and 33.80 mg plant⁻¹ for IC418452) and consortium treatments (32.33 mg plant⁻¹ for Partow and 27.69 mg plant⁻¹ for IC418452) (Fig. S1c).

For seed N concentration, all main effects were significant (*p* < 0.001), with significant interactions for Y × G (*p* < 0.05) and G × F (*p* < 0.01), indicating that genotype effects on seed nitrogen varied by year and fertilizer application. In the second year, the maximum concentration was observed for ‘Partow’ under urea application (Fig. S1d). Seed P concentration showed significant main effects for year (*p* < 0.001), genotype (*p* < 0.001), and fertilizer (*p* < 0.001), but no significant interactions (all *p* > 0.05), implying consistent effects across factor levels (Fig. S1e). Likewise, seed K content (mg g⁻¹) had a highly significant main effect (*p* < 0.001), with no significant interactions (all *p* > 0.05), suggesting that year, genotype, and fertilizer independently influenced seed potassium content, without any combined effects (Fig. S1f).

### Interactive effects on soil nutrient dynamics

The main effects of year (Y), genotype (G), and fertilizer (F) were highly significant for P-CAL (mg/100 g soil) (*p* < 0.001), with significant Y × F (*p* < 0.01) and G × F (*p* < 0.001) interactions, indicating that fertilizer effects on soil phosphorus varied by year and genotype (Table S4). In contrast, Y × G and Y × G × F were not significant (*p* > 0.05). FLNF, KSB, and the control had the lowest P-CAL values (Fig. S2a).

For soil N_min_ (mg/kg soil), genotype and fertilizer effects were significant (*p* < 0.001), whereas year was not (*p* > 0.05). The only significant interaction was Y × F (*p* < 0.001), indicating year-dependent effects of fertilizer on mineral nitrogen, with no genotype-related interactions. Urea was the only fertilizer differing from the others (Fig. S2b).

For soil K (mg/kg soil), all main effects were significant (*p* < 0.001), as were Y × G, Y × F, and G × F interactions (*p* < 0.01), indicating year-dependent genotype and fertilizer effects, and genotype-specific fertilizer responses. The Y × G × F interaction was not significant (*p* > 0.05). In year one, Partow KSB and the control showed the highest soil K concentrations (41.81 and 44.95 mg/kg, respectively), while the lowest values were observed under FLNF and PSB treatments (33.69 mg/kg each). For IC418452, soil K content ranged from 28.94 mg/kg (FLNF and PSB) to 33.98 mg/kg (urea) and 33.86 mg/kg (KSB), with intermediate values under the control (33.24 mg/kg) and consortium (30.09 mg/kg) treatments (Fig. S2c).

### Effects on seed protein and photosynthetic pigments

The main effects of year (Y), genotype (G), and fertilizer (F) were highly significant for seed protein (%) (*p* < 0.001), with significant Y × G (*p* < 0.01) and G × F (*p* < 0.001) interactions, indicating that genotype effects on seed protein varied by year and fertilizer application (Table S5). Y × F and Y × G × F were not significant (*p* > 0.05). In 2022, Partow with urea and microbial consortium application had the highest seed protein content (28.38% and 26.16%, respectively), while IC418452 under urea and consortium showed slightly lower values (19.76% and 20.25%, respectively). The lowest seed protein content was recorded in IC418452 control (16.68%) and Partow control (20.26%) (Fig. S3a).

For chlorophyll a (mg/g FW), all main effects were significant (*p* < 0.001), with Y × F (*p* < 0.001) and G × F (*p* < 0.01) interactions, indicating year- and genotype-dependent fertilizer effects. Y × G and Y × G × F were not significant (*p* > 0.05). Chlorophyll a ranged from 1.34 (Partow control) and 1.17 (IC418452 control) to 2.31 (Partow urea) and 2.12 (IC418452 urea), with consortium treatments showing similarly high values (2.19 and 2.00, respectively) (Fig. S3b).

For chlorophyll b (mg/g FW), all main effects were highly significant (*p* < 0.001), with a significant Y × F interaction (*p* < 0.001), suggesting year-dependent fertilizer effects. Y × G, G × F, and Y × G × F were not significant (*p* > 0.05). In the second year, Partow with urea and microbial consortium application showed the highest chlorophyll b concentrations (1.44 and 1.36 mg/g FW, respectively), followed by IC418452 under the same treatments (1.34 and 1.24 mg/g FW). The lowest chlorophyll b values were found in control plants of both genotypes (Fig. S3c).

Carotenoid concentration (mg/g FW) showed significant effects of year and fertilizer (*p* < 0.001), while genotype and all interactions were not significant (*p* > 0.05), indicating that year and fertilizer had independent effects. In the second year, urea and microbial consortium application yielded the highest carotenoid concentrations, with 0.73 and 0.67 mg/g FW for Partow and 0.71 and 0.64 mg/g FW for IC418452, respectively. At the same time, the lowest values were observed in control treatments (0.26 and 0.29 mg/g FW) (Fig. S3d).

### Regression analysis of root nodulation, grain yield, and soil nutrient

**Fig. 6:**
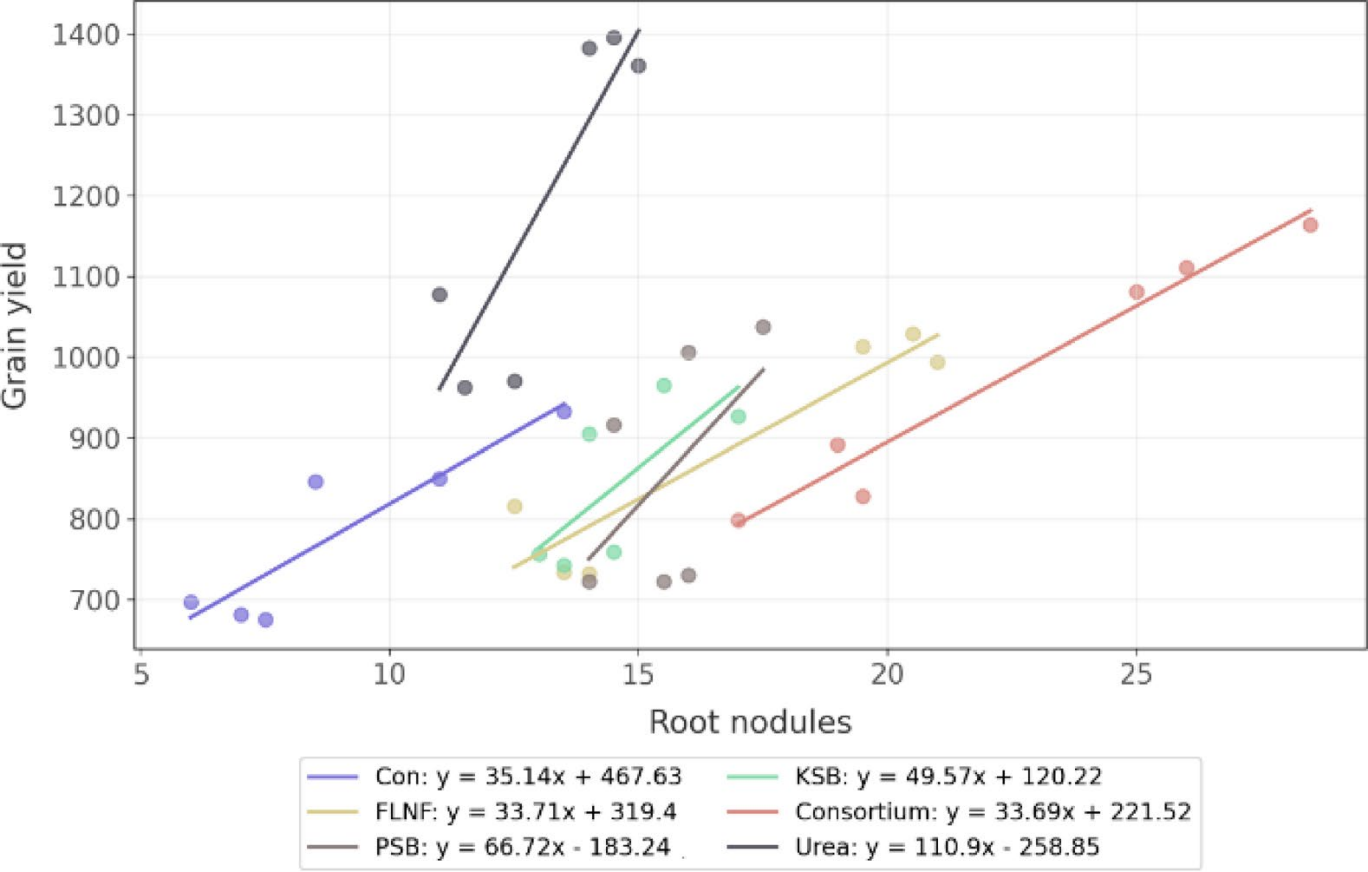
Interaction between the number of root nodules and grain yield across fertilizer treatments. Regression lines and equations are shown for each treatment group. All treatments demonstrate positive associations, with the urea treatment showing the steepest slope (*y* = 110.9 *x* – 258.85), indicating a more substantial effect of root nodules on grain yield under this condition. Treatments include Control (Con), FLNF, PSB, KSB, Consortium, and urea.

The interaction regression analysis between root nodules and grain yield shows a statistically significant positive association (coefficient = 35.14, *p* = 0.018). Increasing root nodules is linked with higher grain yield (Fig. 6). Among the treatments, the urea treatment has a significant negative main effect (coefficient = − 726.47, *p* = 0.040). The significant interaction for the urea treatment (coefficient = 75.76, *p* = 0.010) indicates that this treatment modifies explicitly the relationship between root nodules and grain yield. The positive effect of root nodules on grain yield is even stronger under the urea treatment.

**Fig. 7:**
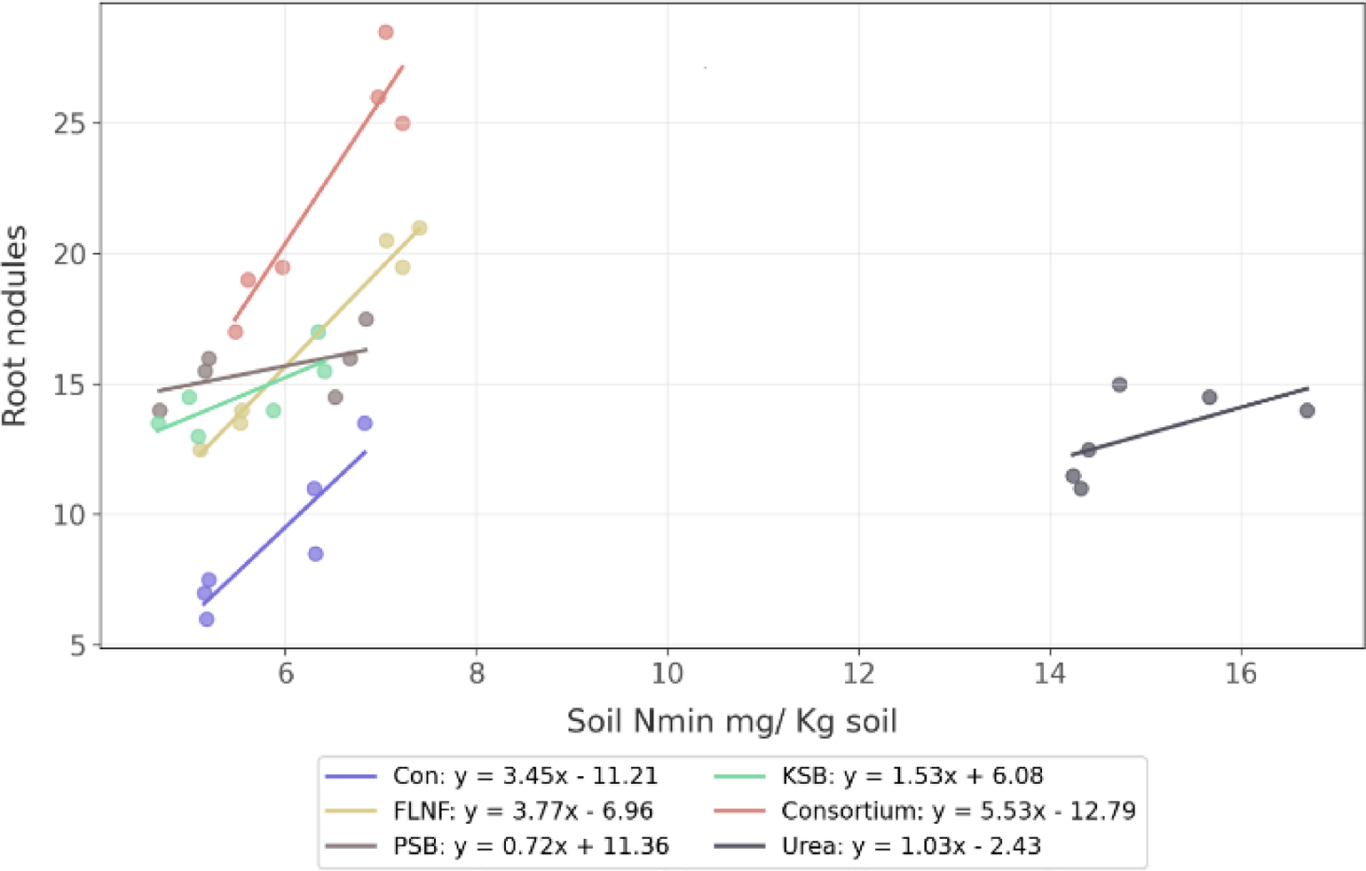
Interaction between the number of root nodules and soil Nmin across fertilizer treatments. Regression lines and equations are shown for each treatment group.

The interaction between soil Nmin and fertilizer treatments is statistically significant for PSB (*p* = 0.010) and urea (*p* = 0.018) treatments, with negative coefficients (− 2.74 and − 2.43, respectively). This confirms that some treatments exhibit negative correlations (Fig. 7). The PSB and KSB treatments display significant positive main effects (coefficients = 22.61 and 17.33, respectively, *p* < 0.01), indicating that these treatments generally promote root nodule formation. About 95.6% of the variance in root nodules (*R*² = 0.956) indicates that fertilizer treatment, soil Nmin, and their interactions strongly predict root nodule formation. The plot shows that the regression lines for the fertilizer treatments have distinctly different slopes: the Control and FLNF show positive relationships, while PSB, KSB, and urea show negative relationships, and the Consortium shows a steeper positive relationship.

**Fig. 8:**
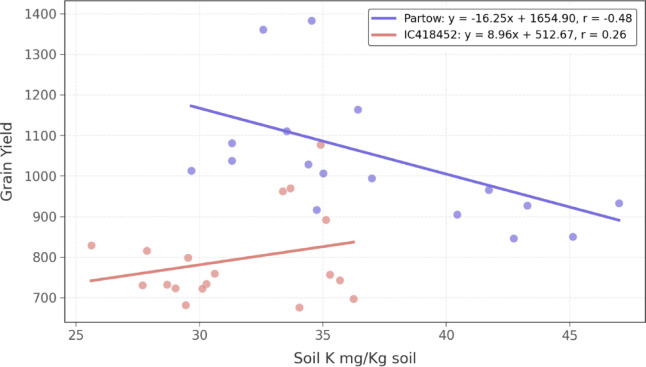
Relationship between soil potassium content (Soil K mg/Kg soil) and grain yield for two mung bean genotypes, Partow and IC418452. The scatter plot shows a negative correlation for Partow (*y* = −16.25 *x* + 1654.90, *r* = −0.48), indicating that higher soil K levels are associated with reduced grain yield. At the same time, IC418452 exhibits a positive correlation (*y* = 8.96 *x* + 512.67, *r* = 0.26), suggesting increased grain yield with higher soil K levels. Data points represent individual measurements for each genotype.

Figure 8 shows a moderate negative correlation (*r* = − 0.48) between soil potassium and grain yield. The regression equation (*y* = − 0.01 *x* + 3.10) indicates that for every 100 mg kg⁻¹ increase in soil potassium, grain yield decreases by approximately 1 unit. The figure also depicts a weak positive correlation (*r* = 0.26) between soil potassium and grain yield in IC418452. The regression equation (*y* = 0.01 *x* + 1.31) indicates that for every 100 mg kg⁻¹ increase in soil potassium, grain yield increases by approximately 1 unit.

## Discussion

There is a greater understanding of microbial bioinoculants, such as PGPR (Plant Growth-Promoting Rhizobacteria). These are increasingly considered the key components of an integrated nutrient-management strategy, a counter to the long-term damaging effects of nutrient imbalances and reducing productivity, and a cost-efficient method to cope with the rising price of fertilizers^32^. The results of this study demonstrate that the type of fertilization has a strong influence on mung bean yield, root morphology, and nutrient uptake in mung beans.

### Influence of climatic conditions on treatment efficacy

Significant year effects and interactions (Tables 3 and 4, S1, S2) underscore the role of climatic variability. Total precipitation from planting to harvest was higher in 2021 (184.38 mm) than in 2022 (84.81 mm). However, 2022 had a more even distribution during key growth phases (Fig. 1), which potentially benefited PGPR by maintaining optimal soil moisture for colonization and activity^9^. PGPR synthesizes hormones such as indole-3-acetic acid (IAA or auxin), cytokinin, and gibberellin, which stimulate root elongation and branching^33,34^. This expands the root surface area, facilitating greater nitrogen absorption from the soil^35^. Strains such as Pseudomonas putida^36^ and Citrobacter sp^37^. produce high levels of IAA, thereby enhancing root development and nutrient efficiency^38^. Biofertilizers (e.g., consortia) enhanced nodulation and plant growth^39^, likely due to the drought tolerance of PGPR and improved root exudation under stress^40^. Higher temperatures may have accelerated bacterial N-fixation but increased urea volatilization^41^, which explains the Y × F interactions (e.g., lower urea efficacy in root volume, Table S2). These findings highlight PGPR’s resilience in variable climates^42^, supporting sustainable mung bean production in arid regions, such as Mashhad.

### Yield, biomass, and growth responses

The combined ANOVA (Table 3) revealed highly significant main effects of fertilizer treatment on biomass, grain yield, 100-seed weight, plant height, and number of pods per plant, which was confirmed by previous research^43,44^. Consistent with expectations, urea application often yielded the highest values for these traits. For instance, the highest biomass was recorded for Partow under urea in the second year (Fig. 2), driven by significant G × F and Y × F interactions. Similarly, grain yield peaked with Partow under urea in the second year (Fig. 3b), influenced by significant Y × G, Y × F, and G × F interactions (Table 3). This aligns with the known role of readily available N in maximizing growth and yield components^45,46^.

Similar to previous findings^47–50^, the microbial consortium significantly enhanced performance compared with the control for these key parameters^28^. While generally yielding less than urea, the consortium treatment achieved plant heights statistically similar to those of urea in some cases (Fig. 3a) and significantly enhanced yield components compared to the control. This supports the efficacy of PGPR in enhancing crop performance^51^, likely through the combined nutrient-enhancing actions of its constituents (FLNF, PSB, KSB)^52^. The significant G × F interactions observed for biomass, grain yield, and pods per plant (Table 3) emphasize that the response magnitude to urea and the consortium varied between the Partow and IC418452 genotypes. The individual biofertilizers (FLNF, PSB, KSB) generally yielded intermediate results, superior to the control but less effective than the consortium or urea, suggesting synergistic benefits within the consortium, consistent with findings in other legumes^53^.

### Root morphology and nodulation dynamics

Root development, significantly influenced by fertilizer treatments (Table 4), showed interesting patterns. Urea application resulted in the largest root area (Fig. 4a) and root volume (Fig. 4c), particularly for the Partow genotype, showing a significant G × F interaction (Table 4). This increased root system size under urea likely supported the higher aboveground biomass observed^54^.

However, the most striking result is related to root nodulation. The microbial consortium treatment resulted in the highest number of nodules per plant (Fig. 4b) and the highest inoculation percentage (Fig. 4d), significantly outperforming all other treatments, including urea. This strongly indicates successful colonization and stimulation of nodulation by the FLNF component, enhancing potential BNF^55^. The significant Y × G × F interaction for nodule number (Table 4) further highlights the complexity, with Partow under the consortium in year 2 showing the peak response (Fig. 4b). The positive correlation between root nodules and grain yield (Fig. 6) suggests that enhanced nodulation directly contributes to higher productivity^56^, likely through improved nitrogen fixation and nutrient assimilation^57^. Notably, urea and consortium treatments exhibited the most substantial increases in nodule formation and yield, with the consortium promoting biological N-fixation for sustainable gains. In contrast, urea may indirectly support nodulation by providing rapid nitrogen availability early in the growth cycle^58^.

Urea treatment showed a negative primary effect on the nodule-yield intercept but a significant positive interaction, indicating it amplifies the positive impact of nodules on yield^59^. This may occur because urea provides immediate nitrogen, reducing early Nitrogen Stress and allowing nodules to focus on long-term fixation^60^, potentially enhancing symbiotic efficiency. Possible reasons include stimulated root growth for improved nodule formation and indirect benefits through enhanced plant vigor^61^. However, excessive nitrogen (N) can suppress nodulation in some legumes^62,63^—here, a balanced application (40 kg/ha) avoided inhibition, promoting yield (up to 38% higher than the control). However, the consortium, FLNF, and control treatments showed a positive relationship between soil Nmin and nodules (Fig. 7), suggesting a reliance on fixation under lower N conditions^64^.

### Nutrient uptake and soil nutrient status

Fertilizer treatments significantly altered plant tissue and soil nutrient concentrations (Tables S5, S4). As expected, urea application resulted in the highest N content in shoots (Fig. S1a) and seeds (Fig. S1d), leading to the highest seed protein percentage, particularly in Partow during the second year. The microbial consortium also significantly increased N concentration and protein content compared to the control, demonstrating the contribution of BNF^65^. However, this was generally less than that of direct urea application.

Evidence for P solubilization was observed in the shoot P content (Fig. S1b), which was significantly higher under PSB and consortium treatments compared to the control (Table S5). This aligns with the hypothesized function of PSB^66^. Available soil P for plants (P-Cal) was also affected, with significant Y × F and G × F interactions, indicating complex dynamics. However, raw PSB and consortium tended towards higher levels than control. Soil K dynamics were also complex, with significant Y × G, Y × F, and G × F interactions. The KSB treatment did not consistently increase plant K uptake but did influence soil K levels, especially in the first year. The contrasting correlations between soil K and grain yield for the two genotypes suggest genotype-specific K utilization or differential responses under varying K availability shaped by the treatments^67^.

### Genotype and year interactions

The study consistently showed Partow outperforming IC418452 in yield and most growth parameters across treatments and years despite IC418452’s noted stress tolerance^30^. The numerous significant G × F interactions (e.g., for biomass, yield, pods, root area, nodules, N content, and protein) firmly establish that the two genotypes responded differently to the fertilizer inputs, reinforcing the need for genotype-specific management recommendations. The significant year effect and the frequent Y × F and Y × G interactions highlight the strong influence of environmental conditions — likely rainfall differences between 2021 and 2022 — on overall performance and the relative effectiveness of different treatments and genotypes^68^.

The results of this study consistently revealed that, despite Partow’s stress tolerance, it outperformed IC418452 in terms of yield and most growth parameters across treatments and years. Genotype differences in soil K response suggest varying K demands—Partow may experience luxury consumption or imbalance at high K, while IC418452 benefits from uptake efficiency^22^. In practice, genotype-tailored plans are essential: lower K for Partow, higher for IC418452, integrated with N/P for synergy^69^.

### Photosynthesis and quality

Enhanced nitrogen by PGPR supports chlorophyll formation, as nitrogen is a core component of the porphyrin ring in chlorophyll molecules. Inoculation with *Azospirillum brasilense* in wheat increases chlorophyll a and b, along with carotenoids like β-carotene and lutein, within days^70^. Photosynthetic pigments (chlorophyll a, b, and carotenoids) were significantly enhanced by urea and the microbial consortium, particularly in the more favorable second year. This improved photosynthetic capacity, likely linked to better N status under these treatments, contributed to the observed biomass and yield gains. The parallel increase in seed protein (Fig. S3a) under urea and the consortium represents a significant quality enhancement^71^.

### Conclusions and implications

Based on significant statistical evidence across multiple parameters, this study demonstrates that urea and a multi–strain PGPR consortium substantially improve mung bean productivity compared to no fertilization. Urea maximized yield, biomass, and N uptake, confirming the effectiveness of chemical N. However, the microbial consortium achieved significant yield improvements, approached the effectiveness of urea in enhancing photosynthetic pigments and protein content, and uniquely excelled in promoting root nodulation, indicating an enhanced BNF potential. This positions the consortium as a viable strategy to reduce reliance on chemical N, aligning with goals for sustainable agriculture. The results highlight synergistic effects within the consortium and reveal critical genotype–specific responses (G × F interactions) and strong environmental modulation (Y effects and interactions). The unexpected enhancement of the nodule–yield relationship under urea warrants further investigation. Future work should optimize PGPR combinations for genotypes like Partow and investigate the long-term impacts on soil health. Future studies should investigate the effects of controlled climate scenarios to elucidate the environmental impacts on PGPR efficacy further.

## Supplementary Information

Below is the link to the electronic supplementary material.

Supplementary Material 1

## Data Availability

The datasets presented in this study are available in an online repository (https://doi.org/10.7910/DVN/V9Z1GO). The additional graphs and tables are in the article/Supplementary Material.
