# Supplementary material for: Optimizing mung bean productivity and root morphology with biofertilizers for sustainable farming
Source: Sci Rep. 2025 Nov 18;15:40333. doi: 10.1038/s41598-025-28815-8 (PMC12627694; doi:10.1038/s41598-025-28815-8)
Supplement: Supplementary file 1 — Supplementary Material 1 [file 41598_2025_28815_MOESM1_ESM.docx]

Table S1. Means (± SE) for biomass and plant growth indicators traits by Year, Genotype, and Fertilizer. (mean values, n = 3 reps, Fisher’s LSD Test, p ≤ 0.05).

| Year | Genotype | Fertilizer | Biomass (Kg/hec) | Grain yield | Weight of 100 grain | Plant height (cm) | Plant pods |
| --- | --- | --- | --- | --- | --- | --- | --- |
| **2021** | IC418452 | Con | 1477.41 ± 16.3 | 657.66 ± 15.1 | 1.96 ± 0.1 | 49.45 ± 2.2 | 21.0 ± 0.5 |
|  | IC418452 | Consor. | 2043.61 ± 46.8 | 759.91 ± 40.0 | 4.03 ± 0.3 | 60.70 ± 2.7 | 21.0 ± 1.1 |
|  | IC418452 | FLNF | 1930.47 ± 206.5 | 698.7 ± 14.89 | 1.9 ± 0.1 | 49.95 ± 1.0 | 20.0 ± 1.5 |
|  | IC418452 | KSB | 1496.53 ± 23.7 | 673.6 ± 9.99 | 2.43 ± 0.2 | 51.62 ± 1.3 | 19.0 ± 1.1 |
|  | IC418452 | PSB | 1774.76 ± 230.6 | 654.9 ± 22.64 | 1.98 ± 0.1 | 50.27 ± 0.9 | 19.0 ± 0.5 |
|  | IC418452 | Urea | 3509.93 ± 53.3 | 887.4 ± 43.92 | 4.9 ± 0.3 | 60.64 ± 2.9 | 24.6 ± 0.8 |
|  | Partow | Con | 1940.38 ± 23.2 | 833.9 ± 28.58 | 3.85 ± 0.2 | 58.89 ± 1.6 | 21.0 ± 0.5 |
|  | Partow | Consor. | 2824.61 ± 103.9 | 962.1 ± 6.12 | 6.30 ± 0.3 | 72.48 ± 4.8 | 28.7 ± 2.0 |
|  | Partow | FLNF | 2540.34 ± 188.5 | 903.6 ± 14.17 | 4.30 ± 0.1 | 59.18 ± 1.3 | 21.7 ± 0.8 |
|  | Partow | KSB | 2429.9 ± 207.5 | 848.30 ± 11.9 | 4.16 ± 0.1 | 58.83 ± 2.4 | 19.0 ± 0.5 |
|  | Partow | PSB | 2462.17 ± 251.9 | 915.67 ± 32.8 | 4.34 ± 0.3 | 61.0 ± 3.4 | 21.3 ± 1.4 |
|  | Partow | Urea | 4790.53 ± 123.1 | 1174.1 ± 30.9 | 7.34 ± 0.3 | 77.0 ± 2.8 | 36.7 ± 1.4 |
| **2022** | IC418452 | Con | 2156.54 ± 150.6 | 712.8 ± 20.6 | 2.88 ± 0.1 | 55.64 ± 0.4 | 20.3 ± 0.8 |
|  | IC418452 | Consor. | 2734.09 ± 140.3 | 920.4 ± 41.7 | 5.48 ± 0.2 | 68.66 ± 0.2 | 31.0 ± 1.7 |
|  | IC418452 | FLNF | 2848.77 ± 119.2 | 823.8 ± 40.6 | 3.75 ± 0.1 | 60.54 ± 1.3 | 23.7 ± 0.8 |
|  | IC418452 | KSB | 2485.86 ± 100.4 | 833.0 ± 18.5 | 3.40 ± 0.1 | 62.62 ± 1.1 | 24.0 ± 1.7 |
|  | IC418452 | PSB | 2750.99 ± 139.9 | 796.6 ± 19.2 | 3.49 ± 0.1 | 60.17 ± 0.3 | 24.7 ± 1.2 |
|  | IC418452 | Urea | 3984.74 ± 155.0 | 1119.8 ± 30.5 | 6.91 ± 0.4 | 76.19 ± 1.2 | 40.0 ± 2.3 |
|  | Partow | Con | 2710.61 ± 146.8 | 919.5 ± 28.1 | 2.81 ± 0.3 | 66.60 ± 2.5 | 19.7 ± 1.4 |
|  | Partow | Consor. | 3755.62 ± 126.0 | 1275.4 ± 42.8 | 6.07 ± 0.3 | 85.89 ± 2.3 | 42.3 ± 1.2 |
|  | Partow | FLNF | 3542.62 ± 28.3 | 1120.8 ± 18.6 | 3.77 ± 0.1 | 74.83 ± 1.1 | 25.3 ± 1.4 |
|  | Partow | KSB | 3059.37 ± 114.9 | 1017.4 ± 23.9 | 3.57 ± 0.2 | 78.02 ± 1.3 | 32.7 ± 0.8 |
|  | Partow | PSB | 3276.69 ± 135.9 | 1058.5 ± 41.4 | 3.73 ± 0.1 | 73.2 ± 1.1 | 24.0 ± 1.5 |
|  | Partow | Urea | 5962.96 ± 54.3 | 1586.9 ± 15.0 | 7.68 ± 0.1 | 90.78 ± 2.9 | 53.7 ± 2.6 |

Table S2. Means (± SE) for root structure and indicators traits by Year, Genotype, and Fertilizer. (mean values, n = 3 reps, Fisher’s LSD Test, p ≤ 0.05).

| Year | Genotype | Fertilizer | Total root area (cm2) | Root length (cm) | Root volume (cm3) | No. of root nodules per plant | Inoculation (%) |
| --- | --- | --- | --- | --- | --- | --- | --- |
| **2021** | IC418452 | Con | 108.35 ± 2.6 | 2564.1 ± 44.9 | 53.58 ± 0.9 | 6.3 ± 0.8 | 61.0 ± 1.6 |
|  | IC418452 | Consor. | 305.52 ± 10.7 | 4434.6 ± 192.9 | 76.48 ± 1.0 | 17.6 ± 0.8 | 79.3 ± 2.0 |
|  | IC418452 | FLNF | 114.521 ± 4.2 | 3448.7 ± 99.2 | 55.28 ± 0.9 | 13.3 ± 0.3 | 71.2 ± 1.3 |
|  | IC418452 | KSB | 147.43 ± 14.1 | 3199.0 ± 67.1 | 55.99 ± 2.4 | 13.3 ± 0.8 | 64.2 ± 3.0 |
|  | IC418452 | PSB | 152.35 ± 16.2 | 3749.0 ± 320.8 | 57.92 ± 0.3 | 14.6 ± 0.8 | 64.8 ± 1.9 |
|  | IC418452 | Urea | 369.4 ± 16.9 | 5579.3 ± 113.7 | 92.52 ± 3.4 | 10.6 ± 0.6 | 71.2 ± 3.2 |
|  | Partow | Con | 138.25 ± 3.5 | 3478.3 ± 217.9 | 70.33 ± 0.9 | 9.6 ± 1.4 | 71.0 ± 1.5 |
|  | Partow | Consor. | 422.31 ± 16.9 | 6685.7 ± 204.1 | 100.4 ± 2.2 | 20.0 ± 0.5 | 83.4 ± 1.6 |
|  | Partow | FLNF | 187.94 ± 10.9 | 5068.3 ± 213.8 | 79.55 ± 0.7 | 16.0 ± 0.5 | 77.7 ± 2.2 |
|  | Partow | KSB | 223.03 ± 11.3 | 4766.4 ± 282.8 | 79.50 ± 3.4 | 13.6 ± 0.8 | 69.5 ± 1.8 |
|  | Partow | PSB | 190.12 ± 8.3 | 4683.7 ± 92.6 | 80.13 ± 0.6 | 14.3 ± 0.8 | 74.2 ± 2.1 |
|  | Partow | Urea | 482.90 ± 18.4 | 8135.0 ± 207.9 | 136.3 ± 7.8 | 12.3 ± 0.8 | 70.6 ± 1.4 |
| **2022** | IC418452 | Con | 93.40 ± 5.5 | 2947.3 ± 166.8 | 58.90 ± 4.0 | 7.3 ± 0.8 | 61.7 ± 1.8 |
|  | IC418452 | Consor. | 277.58 ± 22.3 | 4879.5 ± 86.1 | 92.57 ± 2.4 | 19.3 ± 1.7 | 75.3 ± 1.4 |
|  | IC418452 | FLNF | 192.31 ± 15.3 | 4124.1 ± 246.7 | 77.24 ± 1.5 | 13.3 ± 0.8 | 72.7 ± 0.8 |
|  | IC418452 | KSB | 194.54 ± 16.8 | 4490.5 ± 203.5 | 78.18 ± 1.6 | 14.0 ± 0.5 | 61.9 ± 1.0 |
|  | IC418452 | PSB | 187.21 ± 13.3 | 4238.8 ± 274.5 | 77.36 ± 2.4 | 15.6 ± 0.3 | 69.1 ± 3.2 |
|  | IC418452 | Urea | 348.79 ± 2.1 | 6076.0 ± 228.4 | 108.4 ± 3.6 | 12.6 ± 0.3 | 69.6 ± 2.1 |
|  | Partow | Con | 117.14 ± 11.5 | 3559.6 ± 28.2 | 71.37 ± 4.8 | 12.3 ± 1.4 | 71.4 ± 1.9 |
|  | Partow | Consor. | 328.01 ± 9.2 | 6069.3 ± 262.2 | 108.1 ± 4.1 | 33.0 ± 1.5 | 83.7 ± 1.0 |
|  | Partow | FLNF | 203.01 ± 3.6 | 4640.8 ± 186.5 | 92.7 ± 0.7 | 24.6 ± 1.4 | 81.7 ± 1.0 |
|  | Partow | KSB | 240.16 ± 25.5 | 3717.1 ± 317.8 | 77.73 ± 2.4 | 17.3 ± 0.8 | 74.7 ± 1.7 |
|  | Partow | PSB | 191.25 ± 6.1 | 4664.2 ± 477.3 | 82.39 ± 2.3 | 17.6 ± 0.8 | 77.2 ± 1.7 |
|  | Partow | Urea | 491.03 ± 37.8 | 7407.5 ± 117.2 | 146.9± 2.1 | 16.6 ± 0.8 | 74.8 ± 1.8 |

Table S3. Combined analysis of variance of the main and interaction effects of genotypes and different fertilizers of mung bean shoot and seed nutrients concentration over two years.

|  |  |  | **Shoot** |  |  | **Seed** |  |
| --- | --- | --- | --- | --- | --- | --- | --- |
| S.O.V | df | N g per plant | P mg per plant | K mg per plant | N mg per  g | P mg per  g | K mg per g |
| Year (Y) | 1 | 0.0013* | 7.68*** | 397.48*** | 1.78*** | 3.68*** | 18.30*** |
| Genotype (G) | 1 | 0.0255*** | 12.33*** | 413.21*** | 8.43*** | 7.57*** | 64.55*** |
| Fertilizer (F) | 5 | 0.011*** | 67.6*** | 763.71*** | 2.30*** | 1.75*** | 21.75*** |
| Y × G | 1 | 0.0001 ns | 0.61 ns | 11.66 ns | 0.20* | 0.01 ns | 0.87 ns |
| Y × F | 5 | 0.001* | 1.4*** | 15.64 ns | 0.08 ns | 0.03 ns | 0.21 ns |
| Rep × Year | 4 | 0.0001 ns | 0.19 ns | 32.7 *** | 0.07 ns | 0.03 ns | 1.27 ns |
| G × F | 5 | 0.001* | 0.35 ns | 6.25 ns | 0.17** | 0.03 ns | 0.26 ns |
| Y × G × F | 5 | 0.001* | 0.05 ns | 1.19 ns | 0.06 ns | 0.01 ns | 1.02 ns |
| Residual | 44 | 0.01 | 0.19 | 4.42 | 0.05 | 0.031 | 0.5 |

Table S4. Combined analysis of variance of the main and interaction effects of genotypes and different fertilizers of mung bean soil indicators over two years.

| S.O.V | df | P Cal  mg/100g soil | Soil Nmin  mg/ Kg soil | Soil K  mg/Kg soil |
| --- | --- | --- | --- | --- |
| Year (Y) | 1 | 17.5*** | 0.07 ns | 280.08*** |
| Genotype (G) | 1 | 22.34*** | 39.29 *** | 571.16*** |
| Fertilizer (F) | 5 | 14.94*** | 163.77 *** | 141.48*** |
| Y × G | 1 | 0.17 ns | 0.01 ns | 111.1** |
| Y × F | 5 | 1.30** | 6.13 *** | 31.56** |
| Rep × Year | 4 | 0.18 ns | 0.57 ns | 25.32 ns |
| G × F | 5 | 1.74*** | 0.15 ns | 41.57** |
| Y × G × F | 5 | 0.22 ns | 1.01 ns | 22.05 ns |
| Residual | 44 | 0.32 | 0.59 | 11.96 |

Table S5. Combined analysis of variance of the main and interaction effects of genotypes and different fertilizers of mung bean Physiological responses over two years.

| S.O.V | df | Seed protein(%) | Chlorophyll a (mg/g FW) | Chlorophyll b (mg/g FW) | Carotenoid (mg/g FW) |
| --- | --- | --- | --- | --- | --- |
| Year (Y) | 1 | 61.43*** | 0.54*** | 0.89*** | 0.17*** |
| Genotype (G) | 1 | 426.18*** | 0.25*** | 0.25*** | 0.004 ns |
| Fertilizer (F) | 5 | 61.01*** | 1.38*** | 0.94*** | 0.33*** |
| Y × G | 1 | 11.12** | 0.003 ns | 0.006 ns | 0.004 ns |
| Y × F | 5 | 2.98 ns | 0.05*** | 0.036*** | 0.002 ns |
| Rep × Year | 4 | 3.88 ns | 0.003 ns | 0.002 ns | 0.001 ns |
| G × F | 5 | 16.47*** | 0.019 ** | 0.008 ns | 0.002 ns |
| Y × G × F | 5 | 2.21 ns | 0.003 ns | 0.0004 ns | 0.003 ns |
| Residual | 44 | 1.93 | 0.006 | 0.006 | 0.004 ns |


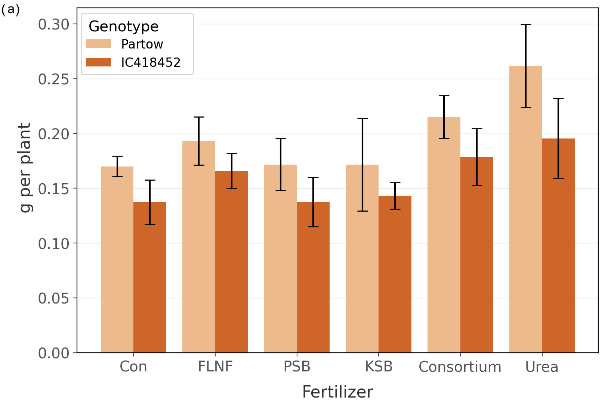

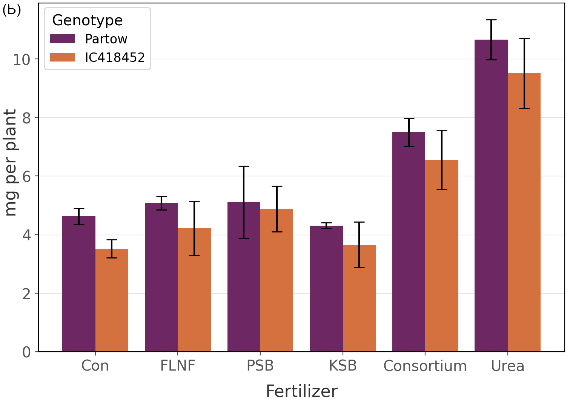


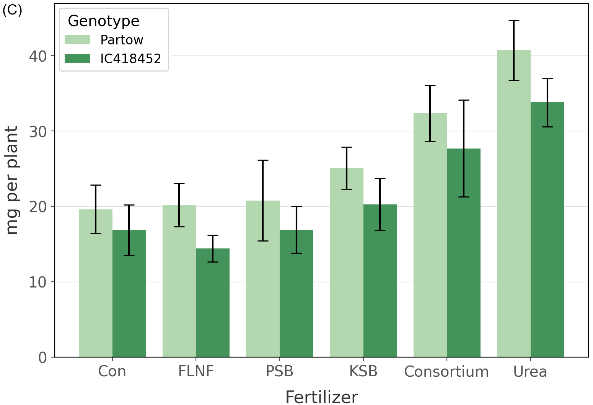

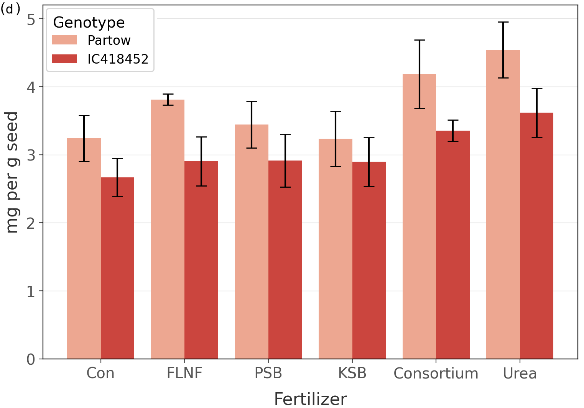

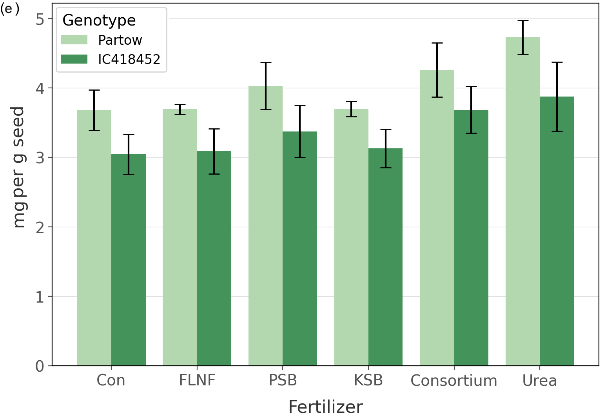

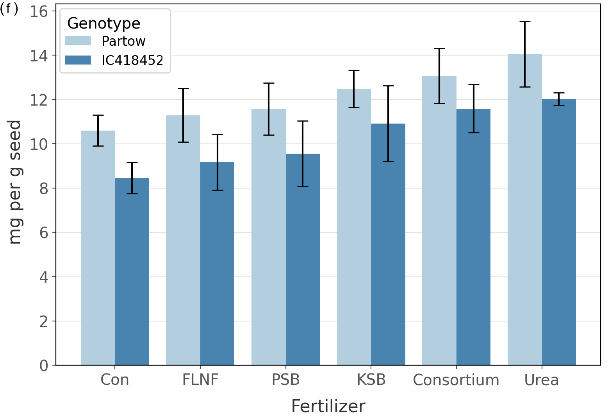


Figure S1. Mean comparison of shoot N content (a), shoot P content (b), shoot K content (c), seed N concentration (d), seed P concentration (e), and seed K concentration (f) of mung bean interaction affected by fertilizers and genotypes under different fertilizers. The error bars on the graph represent a 95% confidence interval (CI).


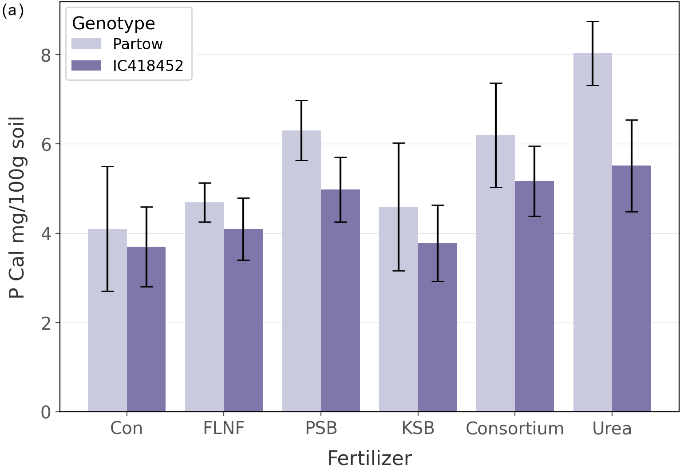

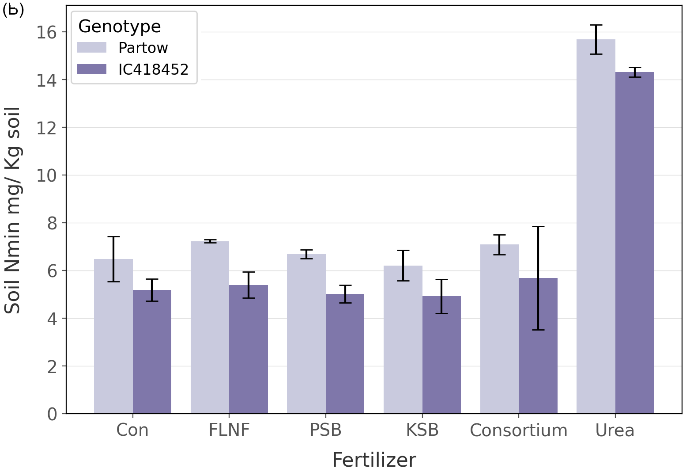


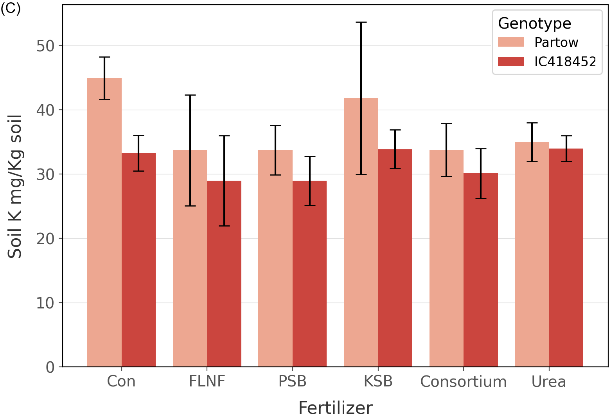


Figure S2. Mean comparison of available soil P (P CAL) (a), soil N min (b), and soil K concentration (c) of mung bean interaction affected by fertilizers and genotypes under different fertilizers. The error bars on the graph represent a 95% confidence interval (CI).


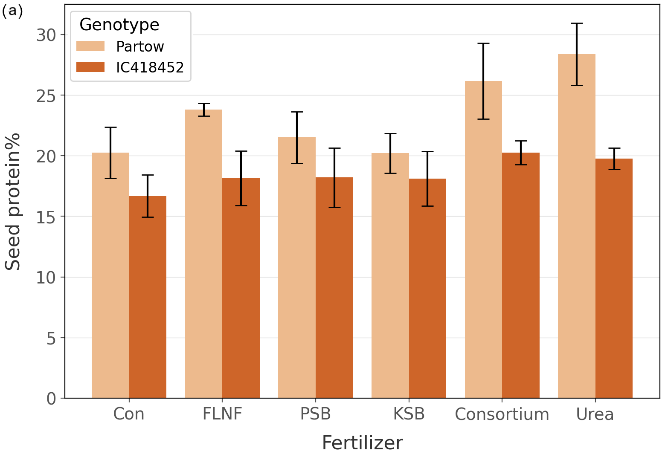

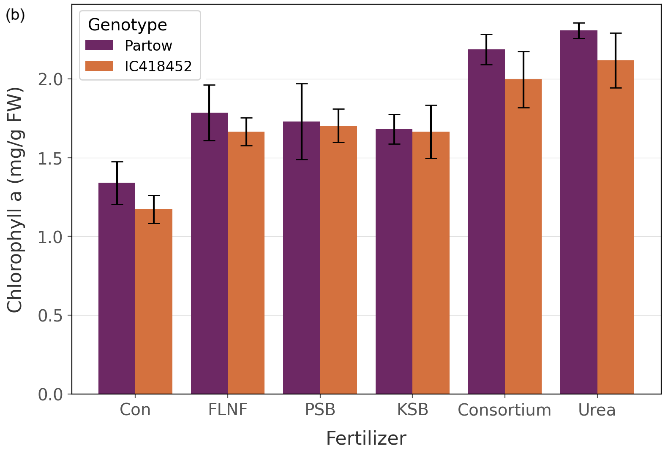


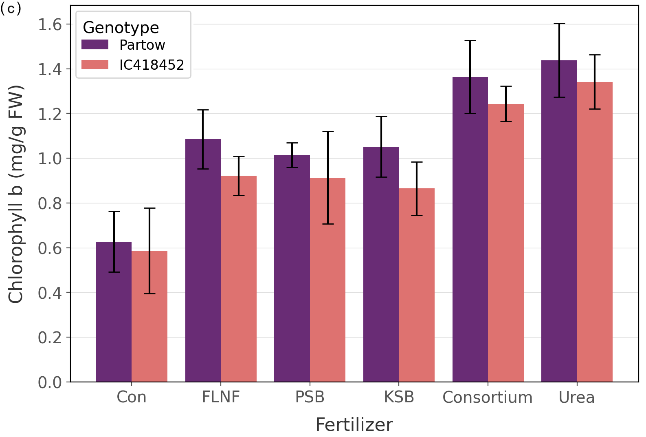

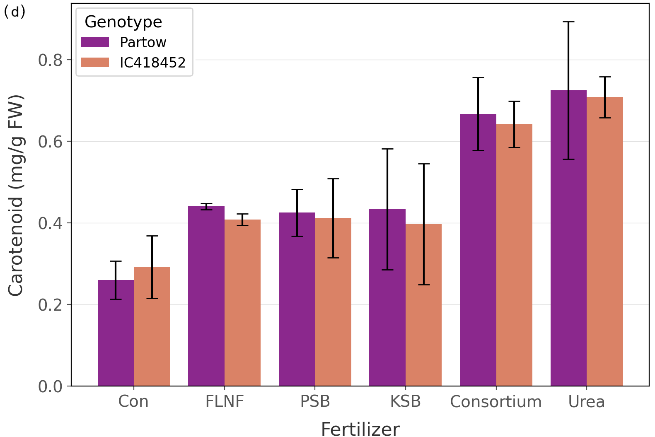


Figure S3. Mean comparison of seed protein (%) (a), chlorophyll a concentration (b), chlorophyll b concentration(c), and carotenoid concentration (d) of mung bean interaction affected by fertilizers and genotypes under different fertilizers. The error bars on the graph represent a 95% confidence in
